# TGF-β regulates phosphorylation and stabilization of Sox9 protein in chondrocytes through p38 and Smad dependent mechanisms

**DOI:** 10.1038/srep38616

**Published:** 2016-12-08

**Authors:** George Coricor, Rosa Serra

**Affiliations:** 1 University of Alabama at Birmingham, Department of Cell, Developmental, and Integrative Biology, Birmingham, Alabama, 35294-0005, USA

## Abstract

Members of the TGF-β superfamily are important regulators of chondrocyte function. Sox9, a key transcriptional regulator of chondrogenesis, is required for TGF-β-mediated regulation of specific cartilage genes. TGF-β can signal through a canonical, Smad-mediated pathway or non-conical pathways, including p38. Here we show that both pathways are activated in chondrocytes after treatment with TGF-β and that TGF-β stabilizes Sox9 protein and increases phosphorylation of Sox9. Mutagenesis of potential serine phosphorylation sites on Sox9 was used to demonstrate that serine 211 is required to maintain normal basal levels of Sox9 as well as mediate increased Sox9 levels in response to TGF-β. The serine 211 site is in a motif that is targeted by p38 kinase. We used siRNA and pharmacological agents to show that p38 and Smad3 independently regulate the phosphorylation and stability of Sox9. Previously, we demonstrated that *Papss2* is a downstream transcriptional target of Sox9 and TGF-β. Here we show that p38 is required for TGF-β-mediated regulation of *Papss2* mRNA. Together the results suggest a new mechanism for TGF-β-mediated gene regulation in chondrocytes via p38 and phosphorylation and stabilization of Sox9. Understanding how TGF-β regulates Sox9 may lead to identification of therapeutic targets for OA.

Articular cartilage is a connective tissue that provides a protective layer for the joints^1^. Injury of this tissue can lead to a common condition called Osteoarthritis (OA)^2^,^3^,^4^. Articular cartilage has limited repair properties. Successful therapeutic approaches to prevent damage or promote repair of cartilage have not been elucidated^5^,^6^. For these reasons, new avenues potentially leading to disease modifying drugs need to be pursued. Previous studies identified important signaling pathways and transcription factors that are affected in OA. One of these, Transforming Growth Factor Beta (TGF-β) plays an important role in cartilage development and homeostasis^7^,^8^. TGF-β signals through serine/threonine kinase receptors known as TGF-β type II (Tgfbr2) and type I (Tgfbr1). When TGF-β ligand binds to Tgfbr2 it recruits Tgfbr1 to form a heteromeric complex. Tgfbr2, a serine/threonine kinase, then phosphorylates Tgfbr1, activating the receptor, which then activates downstream targets^9^,^10^. TGF-β can signal through what are considered canonical and non-canonical pathways^11^. In the canonical pathway, Smad2 or Smad3 are phosphorylated by Tgfbr1. Phospho-Smad2 or 3 (pSmad2/3) then associate with Smad4 and translocate to the nucleus, bind to DNA, and regulate gene expression^10^,^12^. In non-canonical signaling pathways, TGF-β activates MAPK kinase pathways including ERK, JNK, and p38, as well as the Rho-like GTPase, and phosphatidylinositol-3-kinase (PI3K)/AKT pathways^13^. Previously, we showed that mice harboring a dominant negative mutation of Tgfbr2 (DNIIR) exhibited OA-like phenotype^14^. Similar OA-like phenotype was shown in mice deficient in Smad3 and in adult rats with diminished p38 activity^15^,^16^. Over-expression of TGF-β, can help in the repair of articular cartilage, through an increase in Collagen type II (*Col2a*) and Aggrecan (*Acan*) matrix, and inhibition of hypertrophic differentiation^17^,^18^,^19^,^20^. However, increased levels of TGF-β can also lead to osteophyte formation exacerbating the OA phenotype^21^. For this reason, downstream targets of TGF-β that specifically regulate chondroprotective pathways must be identified to develop preventative and reparative therapies.

Sex determining region Y (SRY) Box 9 (Sox9) is an important chondrogenic transcription factor. It regulates formation of embryonic cartilage and is required for post-natal maintenance of the articular cartilage^22^,^23^. Sox9 has been shown to increase *Col2a, Acan*, and *Papss2* expression, as well as decrease expression of matrix degrading proteins like *Mmp9* and *Mmp13*^24^,^25^. Patients with OA exhibited decrease of *SOX9* mRNA^26^. Furthermore, conditional knockout of Sox9 in adult mice causes OA like-phenotype, including loss of *Col2a* and *Acan* expression, and an increase in hypertrophic differentiation^24^,^27^. Over expression of Sox9 in cartilage can lead to repair of cartilage in mice models and *ex-vivo* human OA tissue^28^,^29^. However, it was previously shown that mice over expressing Sox9 have a similar phenotype to mice with loss of Sox9 expression^30^, suggesting that Sox9 must be tightly regulated to function appropriately in cartilage.

TGF-β and Sox9 have similar chondroprotective functions in cartilage. TGF-β and Sox9 are very important for cartilage homeostasis. We previously showed that SOX9 is required for TGF-β1 mediated regulation of *PAPSS2*, an enzyme required for proper sulfation of proteoglycans, in bovine chondrocytes^31^,^32^. For these reasons, we addressed the mechanism of how TGF-β regulates Sox9 protein. We show that treatment with TGF-β results in phosphorylation and stabilization of Sox9 protein in a chondrogenic cell line, ATDC5. Serine 211 was shown to be required for basal and TGF-β-mediated regulation of Sox9 stability. Serine 211 lies within a p38 phosphorylation motif. We then show that TGF-β-mediated phosphorylation and stabilization of Sox9 are dependent on p38 activity. Smad2/3 was also required, independently of p38, for TGF-β-mediated phosphorylation and stabilization of Sox9. The results provide evidence of a novel signaling pathway for TGF-β in chondrocytes.

## Results

### SOX9 protein is phosphorylated and stabilized in response to TGF-β

**Figure 1:**
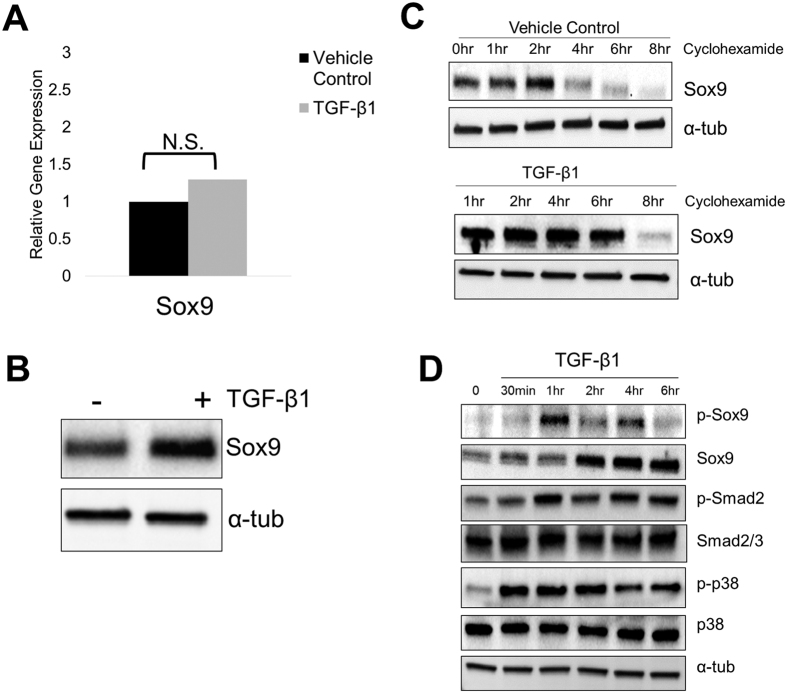
TGF-β1 regulates Sox9 protein. ATDC5 cells were treated with vehicle control or TGF-β1 (5 ng/mL) for 6 hours at which time RNA (**A**) or protein lysates (**B**) were obtained. (**A**) Relative levels of RNA were determined using QRT-PCR. RNA expression was normalized with PPIA. REST software was used for statistical analysis, N.S. denotes Not Significant. (N = 6) (**B**) Immunoblots were used to determine relative levels of Sox9 protein. α-tubulin was used as a normalization control. N = 7. (**C**) ATDC5 cells were pretreated with cyclohexamide (50 ng/mL) for 1 hour. Cells were then treated with vehicle control or TGF-β1 (5 ng/mL). Cell lysates were generated at varying times after treatment and used to determine Sox9 protein levels through western blot, N = 3. (**D**) Western blot analysis of phosphorylated Sox9 (p-Sox9), Sox9, phosphorylated Smad2 (p-Smad2), Smad2/3, phosphorylated p38 (p-p38), and p38 at varying times after treatment with TGF-β1. α-tubulin was used to demonstrate equal loading. N = 3. Western blots were cropped for clarity. Examples of uncropped blots are found in Supplementary Figure S4.

We and others have shown that TGF-β and Sox9 cooperate to regulate expression of specific chondrocyte genes including *Col2a* and *Papss2*^32^,^33^. To determine the mechanism of this cooperation we used the ATDC5 chondrogenic cell line. The ATDC5 cell line is a good *in-vitro* model for the study of cartilage biology. The cells can be utilized for over expression or knockdown studies due to their ease of transfection. We first tested the hypothesis that TGF-β regulates the expression of *Sox9* mRNA and protein in ATDC5 cells (Fig. 1). RNA was isolated from cells that had been treated with TGF-β1 for 6 hours and was used for quantitative real time RT-PCR (QPCR) to determine expression levels of *Sox9* mRNA. Changes in *Sox9* mRNA were not detected after treatment with TGF-β1 (Fig. 1A). Next, protein lysates were collected from control and TGF-β1 treated cells after 6 hours of treatment. Western blot indicated that, although there was no significant change in *Sox9* mRNA levels, Sox9 protein levels were increased (Fig. 1B) suggesting post-translational regulation of Sox9 by TGF-β. To test the hypothesis that TGF-β1 treatment resulted in stability of Sox9 protein in ATDC5 cells, cells were pretreated for 1 hour with cyclohexamide, an inhibitor of protein synthesis. Cells were then treated with vehicle or TGF-β1 and protein lysates were collected over a time course of 0, 1, 2, 4, 6, and 8 hours. We observed prolonged maintenance of Sox9 protein in the TGF-β1 treated cells compared to control cells (Fig. 1C). The results suggest that TGF-β regulates Sox9 protein stability in ATDC5 cells, similar to what we previously observed in bovine articular chondrocytes^32^, supporting the use of ATDC5 cells as a model for cartilage homeostasis in this study.

Previously, TGF-β was shown to stabilize p21; a cell cycle inhibitor; through phosphorylation, a post-translational modification^34^. To determine if TGF-β regulated the phosphorylation of Sox9 in ATDC5 cells, protein lysates were collected from cells that had been treated with TGF-β1 for varying times. Relative phospho-Sox9 (p-Sox9) and Sox9 protein levels were determined by western blot (Fig. 1D). Up-regulation of p-Sox9 was observed by one hour after TGF-β1 treatment and up-regulation of total Sox9 levels was seen later, after 2 hours of treatment. Next, we determined the time course for activation of canonical, Smad-mediated and non-canonical p38-mediated signaling (Fig. 1D). Activation of the Smad pathway was observed by 1 hour after treatment with TGF-β1 as measured by up-regulation of pSmad2. P38 was activated as measured by phosphorylation of p38 (p-p38) by 30 minutes. We then asked if these responses occurred through the Type I receptor. Cells were pretreated with SB431542 (5 μM), a Tgfbr1 inhibitor, or DMSO and then treated with TGF-β1 or vehicle for 2 or 6 hours. The response to TGF-β1 was blocked in the presence of the Type I receptor inhibitor indicating that activation of the canonical Smad2/3 pathway, activation of P38, and up regulation of Sox9 all occurred through TGF-β meditated activation of the Type I receptor (Supplementary Figure S1). Next, we compared the TGF-β response in ATDC5 cells to that of bovine primary chondrocytes. Smad2/3 and p38 were also activated and Sox9 protein regulated by TGF-β in bovine articular chondrocytes with a slightly different time course than what was observed in ATDC5 cells (Supplementary Figure S2). The results support the use of ATDC5 cells as a model for TGF-β-mediated regulation of Sox9 protein, which would be important for cartilage homeostasis.

**Figure 2:**
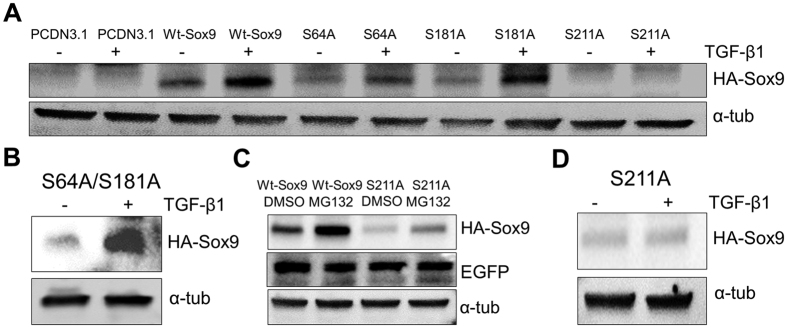
Serine 211 is required for both basal and TGF-β-mediated stabilization of Sox9 protein. (**A**) Expression plasmids (2 μg) containing HA-tagged wild type Sox9 (Wt-Sox9) and serine (S) to alanine (A) point mutants of Sox9 (S64A, S181A, and S211A) were nucleofected into ATDC5 cells. PCDN3.1 was used as a negative control. After 48 hours, cells were treated with TGF-β1 (+, 5 ng/mL) or vehicle (−) for 6 hours. Protein lysates were generated and used for immunblotting with anti-HA antibody. N = 4. (**B**) An expression plasmid containing an S64A/181 A double mutant was nucleofected into ATDC5 cells, after 48 hours cells were treated with TGF-β1 or vehicle control, N = 3. (**C**) Expression plasmids (4 μg) for Wt-Sox9 and S211A were nucleofected in ATDC5 cells. After 48 hours, cells were treated with MG132 (25 ug/mL) or DMSO control for 4 hours. Protein lysates were generated and used in immnoblots with anti-HA antibody. Anti-EGFP antibody was used to insure equal transfection efficiency. N = 3. (**D**) An expression plasmid (4 μg) containing the S211A mutant was nucleofected into ATDC5 cells. After 48 hours cells were treated with TGF-β1 (+) or vehicle control (−) for 6 hours. Lysates were collected for immunblotting with anti-HA antibody. α-tubulin was used to ensure equal loading. N = 3. Western blots were cropped for clarity. Examples of uncropped blots are found in Supplementary Figure S4.

The time course of phosphorylation of Sox9 and subsequent increase in protein levels lead us to the hypothesis that phosphorylation of Sox9 may play a role in stabilizing Sox9 protein. Phosphorylation of Sox9 was previously characterized on serine (S) residues 64, 181, and 211^35^,^36^. To determine if any of these serines are required to regulate stability of Sox9, we generated a series of HA-tagged mutants in which each serine was changed to an alanine (S64A, S181A, and S211A), which cannot be phosphorylated. The mutants were placed into an expression vector and nucleofection was used to transduce each mutant as well as an HA-tagged wild type Sox9 control (Wt-Sox9) into ATDC5 cells. We confirmed that each HA tagged protein was located in the nucleus using immunofluorescent staining (Supplementary Figure S2B). Cells were then either left untreated or treated with TGF-β1 and protein lysates were collected for western blot of the HA tagged proteins (Fig. 2A). As expected, when cells were treated with TGF-β1, HA tagged Wt-Sox9 was up-regulated. S64A and S181A mutants were up-regulated after treatment with TGF-β1 and a S64A/S181A double mutant was also up-regulated by the treatment of TGF-β1 (Fig. 2B), suggesting that Serines 64 and 181 are not required for TGF-β-mediated regulation of Sox9 protein levels. Basal levels of wild type, S64A, and S181A proteins were comparable. In contrast, basal levels of the S211A mutant were barely detectable. We confirmed that the cells were transfected using co-transfection with eGFP and that S211A mRNA was generated (Supplementary Figure S2A), suggesting that S211 is important for regulating the basal stability of Sox9 protein. To further test this hypothesis, Wt-Sox9 or S211A expression vectors were nucleofected into cells, then cells were treated with MG132 proteasome inhibitor or DMSO for 4 hours at which time protein lysates were collected for western blot (Fig. 2C). Treatment with MG132 resulted in an increase in Wt-Sox9 protein compared to DMSO control. Basal levels of S211A were less than Wt-Sox9; however, when treated with MG132 there was an increase in S211A suggesting the S211A protein is synthesized but S211 is required for maintenance of protein levels. Due to the low protein expression of the S211A mutant, we increased the S211A plasmid concentration from 2 μg to 4 μg. Under these conditions, S211A was detectable in the absence of TGF-β1; however, there was still no increase in the S211A mutant protein after treatment with TGF-β1 (Fig. 2D), suggesting Serine 211 is required for both basal and TGF-β-mediated stabilization of Sox9 protein.

### Loss of p38 activity, inhibits TGF-β mediated phosphorylation and up-regulation of Sox9

**Figure 3:**
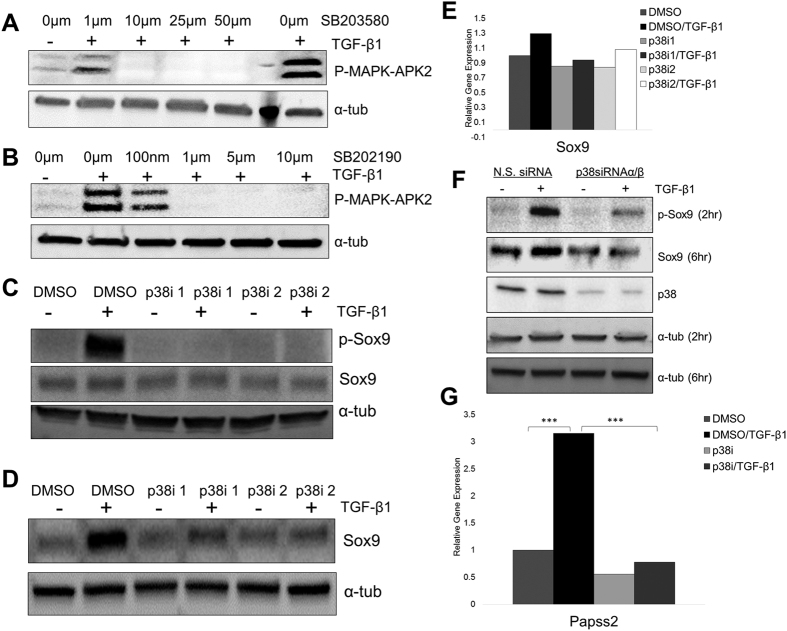
Phosphorylation of Sox9 and up-regulation of Sox9 protein by TGF-β1 is dependent on p38 activity. (**A**,**B**) ATDC5 cells were pretreated with different concentrations of SB203580 (**A**) and SB202190 (**B**). After 24 hours, cells were treated with vehicle control (−) or TGF-β1 (+) for 24 hours. Cell lysates were used in immunblots to measure p-MAPK-APK2 protein levels. N = 3. (**C**) Cells were pretreated with 10 μM SB203580 (p38i 1), 5 μM SB202190 (p38i 2), or DMSO control for 24 hours, and TGF-β1 (+) or vehicle control (−) was added to the cells, which were incubated for an additional 2 hours. Cell lysates were used in immunoblots to detect p-Sox9 protein, N = 4. (**D**) Cell lysates were also collected 6 hours after treatment with TGF-β1 (+) or vehicle control (−) and used to determine Sox9 protein levels, N = 6. (**E**) *Sox9* mRNA levels were measured in cells that were pretreated with p38i 1, p38i 2, or DMSO and then treated with TGF-β1 or vehicle for 6 hours, N = 5. (**F**) ATDC5 cells were transfected with 30 pmol of p38α and p38β siRNA or N.S. siRNA. After 48 hours, cells were treated with TGF-β1 for 2 or 6 hours. Protein lysates were collected and p-Sox9 and Sox9 protein levels were determined by immunoblot, N = 3. (**G**) *Papss2* mRNA levels were measured by QPCR after pretreatment with DMSO or p38i followed by treatment with TGF-β1 or vehicle. mRNA levels were determined relative to DMSO/vehicle treated samples. *PPIA* was used for gene expression normalization. All QPCR data was analyzed using REST software. *** p > 0.001, N = 4. Western blots were cropped for clarity. Examples of uncropped blots are found in Supplementary Figure S4.

Serine 211 in the Sox9 protein is part of a conserved putative p38 phosphorylation motif identified using Kinexus kinase database (Supplementary Table S1). Activation of p38 has been documented as part of one of many non-canonical signaling pathways used by TGF-β^10^ and we showed that p38 was active in ATDC5 cells after treatment with TGF-β1 (Fig. 1D). Furthermore, loss of p38 causes OA-like phenotype in rats^16^. Hence, we tested the hypothesis that TGF-β requires p38 to regulate phosphorylation and stability of Sox9 using two p38 activity inhibitors, sb203580 (p38i1) and sb202190 (p38i2). The inhibitors block downstream phosphorylation of p38 targets including MAPK-APK2. To determine the effective concentrations of the drugs in ATDC5 cells, cells were treated with or without TGF-β1 and varying concentrations of each inhibitor. Protein lysates were collected and the levels of p-MAPK-APK2 were measured (Fig. 3A,B). For subsequent experiments, 10 μM of sb203580 and 5 μM of sb202190 were used since the drugs blocked TGF-β-mediated activation of p38 at these concentrations. Next, cells were pretreated with DMSO or p38i and then treated with vehicle or TGF-β1 for 2 hours. p-Sox9 was measured by western blot (Fig. 3C). In the absence of the inhibitor, treatment with TGF-β1 resulted in an increase in p-Sox9; however, in the presence of each p38i, p-Sox9 was not increased after treatment with TGF-β1. Similar experiments were performed after 6 hours of TGF-β1 treatment to measure the increase in total Sox9 protein levels (Fig. 3D). As expected, in the absence of p38i, treatment with TGF-β1 resulted in an increase in total Sox9 levels; however, in the presence of p38i there was no increase in total Sox9 levels after treatment with TGF-β1. To confirm that p38i did not affect *Sox9* mRNA levels, RNA was isolated from cells treated with vehicle or TGF-β1 after pretreatment with DMSO or p38i and used in QPCR to measure the relative levels of *Sox9* mRNA (Fig. 3E). *Sox9* mRNA was not significantly affected by any of the treatments tested. To avoid any potential off target effects of the p38 inhibitors, we utilized silencing RNA (siRNA) directed to p38α and p38β. The p38 inhibitors, sb203580 and sb202190, inhibit activity of both p38α and p38β. We transfected p38α and p38β siRNA or non-specific siRNA (NS siRNA) into ATDC5 cells, and after 48 hours we treated with TGF-β1 for 2 hours or 6 hours (Fig. 3F). p-Sox9 and Sox9 were not increased by the treatment of TGF-β1 when p38α and p38β were knocked-down. The results indicate that, p38 is required for TGF-β-mediated phosphorylation and stabilization of Sox9 protein.

We previously showed that Sox9 is an important regulator of *Papss2* expression^32^. Here we showed that p38 is required for TGF-β-mediated regulation of Sox9. We would expect that one of the biological consequences of p38 activation by TGF-β and subsequent effects on Sox9 levels would be regulation of *Papss2* mRNA. To test this hypothesis, we isolated RNA from cells that were pretreated with DMSO or p38i then treated with vehicle or TGF-β1 for 6 hours. RNA was used in QPCR assays to measure relative expression of *Papss2* mRNA (Fig. 3G). In the absence of P38i, TGF-β1 treatment resulted in significant up-regulation of *Papss2* mRNA as previously shown^31^,^32^. The p38i alone did not significantly affect *Papss2* expression; however, in the presence of p38i, *Papss2* mRNA levels were not up-regulated in response to TGF-β1. The results indicate that, in addition to Sox9, p38 activity is required for TGF-β-mediated regulation of *Papss2* expression.

### TGF-β-mediated regulation of Sox9 is also dependent on Smad2/3

**Figure 4:**
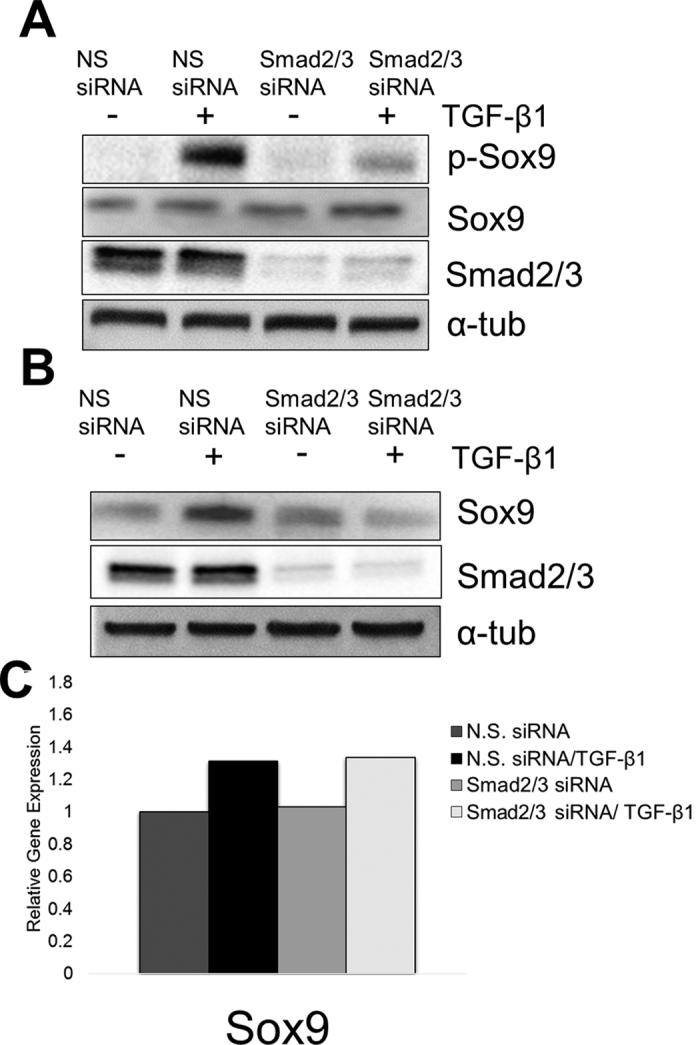
Knockdown of Smad2/3 reduces TGF-β1-mediated phosphorylation and up-regulation of Sox9. (**A**) ATDC5 cells were transfected with 30 pmol non-specific (N.S.) siRNA or mouse Smad2/3 siRNA. After 48 hours, cells were treated with TGF-β1 (5 ng/mL) or vehicle control for 2 hours, then lysates were collected and proteins were immunoblotted using an anti- p-Sox9 antibody, N = 4. (**B**) ATDC5 cells were transfected with siRNAs as indicated. Protein lysates were collected after 6 hours of treatment with TGF-β1. Proteins were used in immunoblots to detect Sox9, N = 4. To ensure equal loading, the membranes were immunoblotted with anti- α-tubulin antibody. Immunoblots of Smad2/3 protein were used to demonstrate knockdown. (**C**) RNA was collected from cells that had been transfected with the indicated siRNA for 48 hours and then treated with TGF-β1 (5 ng/mL) or vehicle for 6 hours. QPCR was used to determine relative *Sox9* mRNA levels. The results are shown relative to vehicle treated cells transfected with N.S. siRNA after normalization with PPIA. REST software was used for statistical analysis. N = 4. Western blots were cropped for clarity. Examples of uncropped blots are found in Supplementary Figure S4.

It was previously shown that TGF-β stabilizes HIF-1 alpha (HIF-1α) through Smad3^37^. In addition, conditional knock out of Smad3 causes OA like phenotype in adult mice^38^ so we tested the hypothesis that Smad2/3 is required for phosphorylation and stabilization of Sox9. We knocked down the expression of Smad2/3 using siRNA and then the cells were treated with TGF-β1 or vehicle. Protein was isolated for western blots at 2 and 6 hours after TGF-β1 treatment (Fig. 4). Knock down of Smad2/3 was confirmed by western blot. After 2 hours of TGF-β1 treatment, cells containing the control NS siRNA demonstrated an increase in p-Sox9 protein. In contrast, p-Sox9 was not up-regulated in cells in which Smad2/3 had been knocked-down (Fig. 4A). Likewise, after 6 hours of treatment, total Sox9 protein was up-regulated in cells treated with the NS siRNA but Sox9 was not up-regulated in Smad2/3 knock-down cells (Fig. 4B). In addition, knock down of Smad2/3 in itself was not sufficient to regulate Sox9 phosphorylation or protein levels, indicating that Smad does not itself regulate Sox9 protein independently of TGF-β. Previously, it was shown that knockdown of Smad3 decreases *Sox9* mRNA levels^33^; however we did not detect any changes in *Sox9* mRNA after knock-down of Smad2/3 (Fig. 4C). The results suggest that, in addition to p38, TGF-β requires Smad2/3 to regulate post-translational phosphorylation and up-regulation of Sox9.

**Figure 5:**
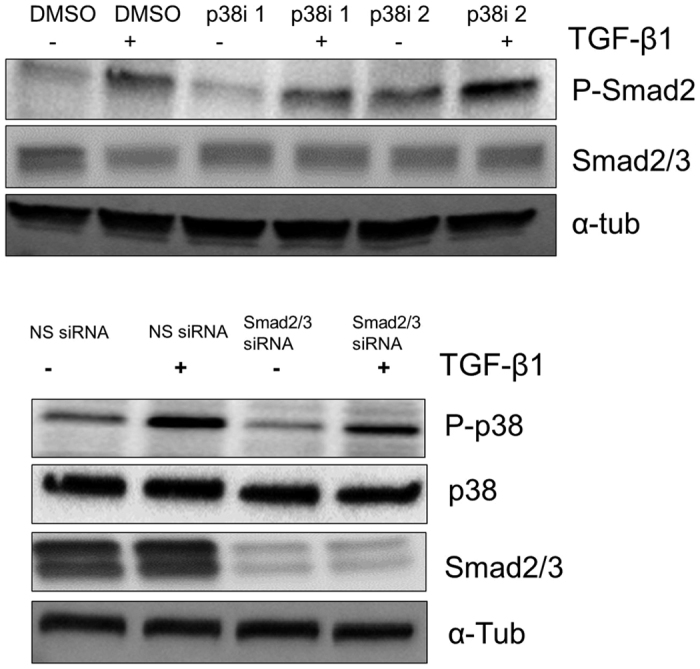
TGF-β1 activates Smad2/3 and p38 independently of each other. (**A**) ATDC5 cells were pretreated with p38 inhibitors (p38i 1 and p38i 2) for 24 hours. The cells were then treated with vehicle (−) or TGF-β1 (+) for 2 additional hours. Protein lysates were collected and used in immunoblots to detect p-Smad. N = 3. (**B**) ATDC5 cells were transfected with siRNA to Smad2/3, after 48 hours the cells were treated with vehicle (−) or TGF-β1 (+) for an additional 2 hours. Protein lysates were used to determine the level of p-p38 via immunoblotting. N = 3. α-tubulin was used as a loading control. Western blots were cropped for clarity. Examples of uncropped blots are found in Supplementary Figure S4.

Previously, cross-talk between p38 and Smad2/3 was demonstrated^20^. Specifically, it was shown that loss of p38 activity results in a decrease in phosphorylation of Smad2/3 after treatment with TGF-β^39^. This raises the possibility of cross-talk between p38 and Smad2/3 in post-translational regulation of Sox9. Specifically, p38 could regulate TGF-β-mediated activation of Smad or Smad could be required for TGF-β-mediated activation of p38. To test this hypothesis, cells were pretreated with DMSO or the p38 inhibitors described above, then cells were treated with vehicle or TGF-β1 for 2 hours, and activation of Smad was measured as levels of pSmad2 on Western blot (Fig. 5A). In the presence of DMSO, p-Smad2 was up-regulated after treatment with TGF-β1, as expected. P-Smad2 was also up-regulated after treatment with TGF-β1 in the presence of each of the p38 inhibitors. The results indicate that p38 activity is not required for activation of canonical Smad signaling in response to TGF-β. Next, cells that expressed NS siRNA or siRNA to Smad2/3 were treated with TGF-β1 for 2 hours and activation of p38 was measured as the up-regulation of p-p38 by Western blot (Fig. 5B). In the presence of the NS siRNA, p-p38 levels were increased after treatment with TGF-β1, as previously shown. P38 was also activated after treatment with TGF-β1 in cells in which Smad2/3 had been knocked-down. The results indicate that canonical Smad signaling is not required for TGF-β- mediated activation of p38 and suggest that p38 and Smad2/3 regulate phosphorylation and stabilization of Sox9 independently.

## Discussion

TGF-β and SOX9 play an important role in the development, maintenance, and protection of articular cartilage^14^,^29^,^40^,^41^. In this study, we show that treatment of ATDC5 cells with TGF-β1 results in stabilization of Sox9 protein (Fig. 1A,C). We then began to determine the mechanism of how TGF-β stabilizes Sox9 by showing that Serine 211 is required for maintenance of basal levels of Sox9 as well for TGF-β-mediated up-regulation of Sox9 protein (Fig. 2A,C,D). Furthermore, Serine 211 resides in a conserved p38 kinase target motif and we show that p38 is required for TGF-β-mediated regulation of Sox9 protein. Smad2/3 was also required for TGF-β to regulate Sox9 protein; however, Smad2/3 and p38 acted independently of each other. Elucidating the mechanism of TGF-β-mediated regulation of Sox9 protein levels may help to identify therapeutic targets for OA.

The most well-characterized mechanism of TGF-β action in cartilage is through direct transcriptional regulation of important cartilage genes, most commonly through Smad transcription factors^12^. In this report we describe a novel mechanism in which TGF-β acts to post-translationally regulate Sox9 protein. Sox9 is an important transcriptional regulator of chondrocyte function, and yet not much is known about how Sox9 protein levels are regulated in cartilage. We show that TGF-β1 up-regulated Sox9 phosphorylation, a post-translational modification (Fig. 1D and S1D), in both ATDC5 cells and bovine articular chondrocytes. Phosphorylation has been shown to play an important role in stability of transcription factors including RUNX2, and ATF2^42^,^43^. Furthermore, TGF-β was shown to stabilize the cell cycle inhibitor p21 through phosphorylation^34^. In both ATDC5 cells and bovine articular chondrocytes, phosphorylation of Sox9 by TGF-β occurred before up-regulation and stabilization of total Sox9 protein levels (Fig. 1D and S1). We hypothesized that TGF-β acts through phosphorylation of Sox9 to regulate its stability. To address this, we generated mutants of three serine residues that were previously reported to be phosphorylated on Sox9. Previous studies indicated that serine 64 and 181 were important for the transcriptional activity of Sox9^35^, but protein stabilization was not addressed. In this report, we show that serine 64 and 181 are not required for maintenance of Sox9 protein levels, or for TGF-β-mediated up-regulation of Sox9 (Fig. 2). In contrast, basal levels of the Sox9 serine 211 mutant were reduced and the serine 211 mutant was not up-regulated by treatment with TGF-β1 (Fig. 2). The results suggest that serine 211 is somehow required for TGF-β to regulate the stability of Sox9. Although we show that Serine 211 is important for maintaining Sox9 protein levels and regulation by TGF-β, we do not know if this is directly through phosphorylation at this site. The findings that serine 211 lies within a p38 phosphorylation motif and that p38 is required for TGF-β to increase Sox9 levels are supportive of the hypothesis. Interestingly enough, S211 is highly evolutionarily conserved (Supplementary Table S1), thus it is reasonable to propose that this site plays an important role in regulating Sox9 whether through phosphorylation or other mechanisms.

Serine 211 lies within a conserved p38 kinase target domain while serine 64 and 181 are known to be targeted by cAMP dependent protein kinase A^35^. These sites have been shown to play a role in transcriptional activity of Sox9^35^. TGF-β can act through several signaling pathways. The most well characterized signaling pathway, so-called canonical pathway, utilizes Smad transcription factors^10^. However, non-canonical TGF-β signaling pathways exist including those that activate p38 kinase^44^. The p38 pathway was shown to be involved in cartilage development and homeostasis^45^. Specifically, loss of p38 activity can lead to development of OA phenotype in adult rats^16^. We observed that inhibition of p38 activity leads to inhibition of TGF-β-mediated phosphorylation and up-regulation of Sox9 (Fig. 3C,D,F). p38 was previously shown to regulate *Sox9* mRNA levels in primary human articular chondrocytes in the presence of cyclohexamide^46^. We did not observe changes in *Sox9* mRNA in the presence of p38 inhibitors (Fig. 3E). Our results suggest the block to TGF-β-mediated up-regulation of Sox9 protein in the presence of p38i was post-translational. p38 could directly phosphorylate Sox9 or it could act indirectly to regulate other proteins that could then act on Sox9.

Previously, we showed that TGF-β acts through Sox9 to regulate expression of the *Papss2* gene^31^,^32^. Papss2 enzyme is important in regulating sulfation of the glycosamino glycans on proteoglycans in the cartilage^47^. We tested the hypothesis that the loss of p38 activity would also block TGF-β-mediated expression of *Papss2* mRNA, presumably through its effects on Sox9 protein levels. We showed that in the presence of p38 inhibitor, there was a significant attenuation in TGF-β-mediated stimulation of *Papss2* mRNA (Fig. 3G). We hypothesize that TGF-β activates p38, which regulates Sox9 protein levels, which regulate *Papss2* mRNA expression.

Activation of Smad2/3 defines the canonical TGF-β signaling pathway. Smad2/3 mediates TGF-β-regulated stability of HIF-1α through transcriptional inhibition of prolyl hydroxylase enzyme 2 (PHD2)^37^. We showed that knockdown of Smad2/3 blocks TGF-β-mediated phosphorylation and stability of Sox9 protein (Fig. 4A,B) suggesting that, in addition to p38, Smads are also required for TGF-β to up-regulate Sox9 protein levels. Smad2/3 signaling plays an important role in cartilage development and homeostasis, and loss of Smad3 leads to loss of cartilage proteoglycans and an increase in hypertrophic differentiation of chondrocytes^48^,^49^. Specifically how Smad2/3 functions to stabilize Sox9 protein is not known. We hypothesize that Smad2/3 may bind to Sox9 directly as was previously shown for regulation of *Col2a* expression^33^. Alternatively, Smad2/3 could regulate additional kinases or phosphatases that would regulate Sox9 protein levels.

Evidence of cross talk between Smads and p38 has been controversial, some studies previously demonstrated that loss of p38 activity decreased Smad2/3 nuclear localization in the presence of TGF-β^50^, and knockout of Smad3 showed decreased phosphorylation of p38 by TGF-β^49^. While other studies have shown that inhibition of p38 was not required to phosphorylate Smad2^51^, and over-expression of dominant negative Smad3 did not inhibit TGF-β mediated activation of p38^52^. We report that p38 and Smad2/3 act independently to regulate Sox9 (Fig. 5A,B).

This study provides evidence of a novel signaling pathway for TGF-β in cartilage that involves post-translational stabilization of Sox9 protein through Smad2/3 and p38 signaling pathways. There are currently no drugs available to cure or prevent OA. To that end, determining the molecular mechanisms of how TGF-β and Sox9 promote the cartilage phenotype may lead to the identification of targets that can be utilized against OA.

## Methods

### Cell Culture

ATDC5 cells were cultured in medium composed of 1:1 mixture of Dulbecco’s modified Eagle’s medium and nutrient mixture F-12 (DMEM/F12; Thermo Fisher Scientific), 5% heat-inactivated fetal bovine serum (hi-FBS, Thermo Fisher Scientific), 1% L-glutamine (L-glut) and 1% penicillin-streptomycin (pen-strep) (Thermo Fisher Scientific) at 37 °C in humidified 5% CO2 incubator^53^. Bovine Articular Chondrocytes were isolated from 1–2 year old bovine metacarpophalangeal joints, and then incubated overnight at 37 °C in PBS containing 2 mg/mL collagenase D (Roche) and 1% pencillin-streptomycin (Thermo Fisher Scientific). Then the digested tissue was subjected through filtration to obtain chondrocytes. After filtration, the cells were placed in culture medium containing DMEM, 10% hi-FBS, 1% L-glut, and 1% pen-strep. Then placed in micromass, and each micormass contained 2 × 10^5^ cells. Micromass cultures were then incubated overnight at 37 °C to attach to the culture dishes. Next day, DMEM was added containing 0.5% hi-FBS, 1% pen strep, 1% L-glut, and 50 μg/mL sodium L-ascorbate^31^,^54^. For ATDC5 cells, 5 ng/mL of TGF-β1 (R&D Systems) was added 24 hours after platting, while the bovine articular chondrocytes were treated with TGF-β1 after 48 hours of platting. Cells were pretreated with p38 inhibitors SB203580 (Abcam), SB202190 (Abcam), or DMSO control for 24 hours before treatment with TGF-β1. Cells were pretreated with proteasomal inhibitor MG132 (Abcam) for 4 hours. ATDC5 cells were treated with 50 μg/mL of cyclohexamide (Fisher Scientific) or DMSO control for 1 hour, then treated with TGF-β1 or vehicle control. ATDC5 cells were pretreated with 5 μM SB431542 (Tocris Bioscience) for 2 hours, then treated with TGF-β1 or vehicle control for 2 or 6 hours.

### Quantitative Real Time PCR

Cells were lysed using TRIzol Reagent (Thermo Fisher Scientific), and total RNA was extracted using Direct-zol RNA MiniPrep kit (Zymo Research). QuantiFast SYBR Green RT-PCR Kit (Qiagen) was used for quantitative real time RT-PCR (QPCR). QPCR was performed using the Roche LightCycler 480 following the manufacturer’s protocol. Quantification of relative mRNA levels between experimental groups was determined using the Relative Expression Software Tool (REST) 2009 (QIAGEN)^55^. REST analysis software is used to determine statistical differences in gene expression across multiple biological replicates. A biological replicate is defined as a separate experiment from a different passage of cells, and all samples for each experiment had three technical replicates. REST analyzes nonparametric gene expression data using a pair-wise fixed reallocation randomization test and includes PCR efficiency corrections. REST output includes the normalized fold difference in gene expression, 60% and 95% confidence intervals, and statistical call. *G*ene expression was normalized using Peptidylprolyl Isomerase A (*PPIA*)^56^. Primer sequences are listed in Table S2. All primers spanned an exon-exon junction.

### Western Blot

ATDC5 cells or Bovine articular chondrocytes were washed with ice-cold PBS, then RIPA buffer containing protease and phosphatase inhibitors was added (Roche). 30–50 μg of protein lysates were separated by electrophoresis on 4–20% polyacrylamide gels (Bio-Rad Laboratories). Then the gels were transferred onto a polyvinylidene fluoride (PVDF) membranes (Bio-Rad Laboratories) using Trans-Blot Turbo Transfer System (Bio-Rad Laboratories). PVDF membranes were blocked for 1 hour with 3% Blotto non-fat dry milk (Santa Cruz Biotechnology) or 3% Bovine Serum Albumin (BSA) (Fisher Scientific). PVDF membranes were probed overnight using the following primary antibodies anti-SOX9 primary antibody (1:1000, Santa Cruz Biotechnology), anti-phospho-Sox9 (1:1000, Abcam), anti-Smad2/3 (1:2000, Cell Signaling), anti-phospho-Smad2 (1:1000, Cell Signaling), anti-p38 (1:1000 Cell Signaling), anti-phospho-p38 (1:1000, Cell Signaling), anti-phospho-Mapk-Apk2 (1:1000, Cell Signaling), anti-HA-Tag (1:1000, Cell signaling), Anti-EGFP (1:1000, Abcam), and anti-α-tubulin (1:2500, Rockland Immunochemicals Inc.) was used as loading control. After overnight incubation, PVDF membranes were washed three times with Tris buffer saline solution containing 0.1% tween 20, and horseradish-peroxidase conjugated secondary antibody (1:2000, Cell Signaling) was added for 1 hour. All blots were imaged using ChemiDoc MP System (Bio-Rad Laboratories). Blots were cropped to make figures. Examples of uncropped blots for each antibody used are shown in Supplementary Figure S4. Overexposing or high contrasting of blots was not used.

### Mutagenesis

Mutant HA-Tagged Sox9 plasmids (S64A, S181A, S211A) were made using QuikChange Lightning Multi-Site-Directed Mutagenesis Kit (Agilent Technologies) as per manufacturer’s instruction. Mutations were confirmed by Sanger sequencing.

### Silencing RNA knockdown

30 pmol of mouse *Smad2*/*3* silencing RNA (siRNA) (Santa Cruz), or p38α siRNA (Cell Signaling), p38β siRNA (Cell Signaling), or non-specific control siRNA (Santa Cruz, Cell Signaling) were transfected into ATDC5 cells using Lipofectamine RNAi (Thermo Fisher Scientific) as per manufacturer’s protocol. Then after 48 hours of transfection, cells were treated with TGF-β1 or vehicle control. FITC-conjugated control siRNA (Santa Cruz) was used to test for transfection efficiency, approximately 80–90% of cells were transfected with siRNA.

### Nucleofection

All plasmids were prepared using Zyppy Plasmid Miniprep Kit (Zymo Research). For nucleofection, 1 × 10^6^ ATDC5 cells per mL were placed in tubes and spun at 100 g for 10 minutes. Then cells were re-suspended in the SG cell line 4D-Nucleofector solution (Lonza) containing 2 μg–4 μg of either PCDN3.1 vector plasmid, Wt-Sox9, S64A, S181A, S64/181A, or S211A plasmid. An eGFP plasmid was co-nucleofected with each sample as a control for transfection efficiency. Then the mixture was transferred into a 100 μl nucleocuvette (Lonza) and placed in 4D-Nucleofector (Lonza) for nucleofection. Next, 100 μl of pre-warmed DMEM/F12 (Thermo Fisher Scientific) was added to each nucleocuvette, and incubated for 10 minutes at room temperature. After 10 minutes, the cells were added to pre-warmed ATDC5 cells medium, and incubated at 37 °C for 48 hours. Cells were treated with TGF-β1 (5 ng/mL) or vehicle control for indicated times, then cells were lysed for use in western blot or QPCR.

### Immunofluorescence

HEK 293 cells were platted on chamber slides (Thermo Fisher Scientific). After 24 hours, cells were transfected with Wt-Sox9, S64A, S181A, and S211A plasmid using ViaFect Transfection reagent (Promega) as per manufacturing instructions. After 24 hours of transfection, cells were washed with phosphate buffer saline (PBS), then covered with 4% formaldehyde diluted in PBS. After 15 minutes of fixation at room temperature, cells were washed with PBS, then blocking buffer containing 5% normal goat serum (Sigma-Aldrich) and 0.3% Triton X-100 (Fisher Scientific) in PBS was placed on the cells for 1 hour. Next, cells were washed with PBS and primary antibody solution having Anti-HA-Tag (1:500, Cell Signaling), 1% BSA, 0.3% Triton X-100 were added to the cells and incubated overnight at 4 °C. The next day, cells were washed with PBS, then cells were incubated with secondary antibody Alexa Flour 488 (1:1000, Thermo Fisher Scientific) for 1 hour. After 1 hour incubation, the cells were washed with PBS and VECTASHIELD HardSet Antifade Mounting Medium with DAPI (Vector Labs) with coverslips were placed on the slides.

## Additional Information

**How to cite this article**: Coricor, G. and Serra, R. TGF-β regulates phosphorylation and stabilization of Sox9 protein in chondrocytes through p38 and Smad dependent mechanisms. *Sci. Rep.* **6**, 38616; doi: 10.1038/srep38616 (2016).

**Publisher's note:** Springer Nature remains neutral with regard to jurisdictional claims in published maps and institutional affiliations.

## Supplementary Material

Supplementary Information

## References

[b1] Sophia Fox A. J., Bedi A. & Rodeo S. A. The Basic Science of Articular Cartilage: Structure, Composition, and Function. Sports Health 1, 461–468, doi: 10.1177/1941738109350438 (2009). 23015907 PMC3445147

[b2] Heinegård D. & Saxne T. The role of the cartilage matrix in osteoarthritis. Nat Rev Rheumatol 7, 50–56, doi: 10.1038/nrrheum.2010.198 (2011). 21119607

[b3] Lories R. J. & Luyten F. P. The bone-cartilage unit in osteoarthritis. Nat Rev Rheumatol 7, 43–49, doi: 10.1038/nrrheum.2010.197 (2011). 21135881

[b4] Dreier R. Hypertrophic differentiation of chondrocytes in osteoarthritis: the developmental aspect of degenerative joint disorders. Arthritis Res Ther 12, 216, doi: 10.1186/ar3117 (2010). 20959023 PMC2990991

[b5] Li Y., Wei X., Zhou J. & Wei L. The Age-Related Changes in Cartilage and Osteoarthritis. BioMed Research International 2013, 12, doi: 10.1155/2013/916530 (2013). PMC3736507 23971049

[b6] Pelletier J.-P., Martel-Pelletier J. & Abramson S. B. Osteoarthritis, an inflammatory disease: Potential implication for the selection of new therapeutic targets. Arthritis & Rheumatism 44, 1237–1247, doi: 10.1002/1529-0131(200106)44:6<1237::AID-ART214>3.0.CO;2-F (2001). 11407681

[b7] Blaney Davidson E. N., van der Kraan P. M. & van den Berg W. B. TGF-β and osteoarthritis. Osteoarthritis and Cartilage 15, 597–604, doi: 10.1016/j.joca.2007.02.005 (2007). 17391995

[b8] Shen J., Li S. & Chen D. TGF-β signaling and the development of osteoarthritis. Bone Research 2, 14002, doi: 10.1038/boneres.2014.2 (2014). 25541594 PMC4274935

[b9] Moses H. & Serra R. Regulation of Differentiation by TGF-beta. Current Opinion in Genetics and Development 6, 581–586 (1996). 8939725 10.1016/s0959-437x(96)80087-6

[b10] Massague J., Blain S. & Lo R. TGFbeta signaling in growth control, cancer, and heritable disorders. Cell 103, 295–309 (2000). 11057902 10.1016/s0092-8674(00)00121-5

[b11] Wrana J., Attisano L., Wieser R., Ventura F. & Massague J. Mechanism of activation of the TGF-beta receptor. Nature 370, 341–347 (1994). 8047140 10.1038/370341a0

[b12] Serra R. & Chang C. TGF-beta signaling in human skeletal and patterning disorders. Birth Defects Research (Part C) 69, 333–351 (2003). 10.1002/bdrc.10023 14745974

[b13] Akhurst R. J. & Hata A. Targeting the TGF[beta] signalling pathway in disease. Nat Rev Drug Discov 11, 790–811 (2012). 23000686 10.1038/nrd3810 PMC3520610

[b14] Serra R. . Expression of a truncated, kinase-defective TGF-beta type II receptor in mouse skeletal tissue promotes terminal chondrocyte differentiation and osteoarthritis. J Cell Biol 139, 541–552 (1997). 9334355 10.1083/jcb.139.2.541 PMC2139797

[b15] Yang X. . TGF-beta/Smad3 signals repress chondrocyte hypertrophic differentiation and are required for maintaining articular cartilage. J Cell Biol 153, 35–46 (2001). 11285272 10.1083/jcb.153.1.35 PMC2185521

[b16] Prasadam I. . Inhibition of p38 pathway leads to OA-like changes in a rat animal model. Rheumatology 51, 813–823, doi: 10.1093/rheumatology/ker360 (2012). 22240502

[b17] Venkatesan J. K. . rAAV-mediated overexpression of TGF-β stably restructures human osteoarthritic articular cartilage *in situ*. Journal of Translational Medicine 11, 1–14, doi: 10.1186/1479-5876-11-211 (2013). 24034904 PMC3847562

[b18] Zhang X. . Primary murine limb bud mesenchymal cells in long-term culture complete chondrocyte differentiation: TGF-β delays hypertrophy and PGE2 inhibits terminal differentiation. Bone 34, 809–817, doi: 10.1016/j.bone.2003.12.026 (2004). 15121012

[b19] Baugé C., Cauvard O., Leclercq S., Galéra P. & Boumédiene K. Modulation of transforming growth factor beta signalling pathway genes by transforming growth factor beta in human osteoarthritic chondrocytes: involvement of Sp1 in both early and late response cells to transforming growth factor beta. Arthritis Research & Therapy 13, 1–13, doi: 10.1186/ar3247 (2011). PMC3241367 21324108

[b20] Watanabe H., de Caestecker M. P. & Yamada Y. Transcriptional cross-talk between Smad, ERK1/2, and p38 mitogen-activated protein kinase pathways regulates transforming growth factor-beta-induced aggrecan gene expression in chondrogenic ATDC5 cells. The Journal of biological chemistry 276, 14466–14473, doi: 10.1074/jbc.M005724200 (2001). 11278290

[b21] Scharstuhl A. . Inhibition of Endogenous TGF-β During Experimental Osteoarthritis Prevents Osteophyte Formation and Impairs Cartilage Repair. The Journal of Immunology 169, 507–514, doi: 10.4049/jimmunol.169.1.507 (2002). 12077282

[b22] Bi W., Deng J. M., Zhang Z., Behringer R. R. & Crombrugghe B. Sox9 is required for cartilage formation. Nat Genet. 22, doi: 10.1038/8792 (1999). 10319868

[b23] Henry S. P., Liang S., Akdemir K. C. & de Crombrugghe B. The postnatal role of Sox9 in cartilage. Journal of bone and mineral research: the official journal of the American Society for Bone and Mineral Research 27, 2511–2525, doi: 10.1002/jbmr.1696 (2012). PMC3502666 22777888

[b24] Akiyama H., Chaboissier M.-C., Martin J. F., Schedl A. & de Crombrugghe B. The transcription factor Sox9 has essential roles in successive steps of the chondrocyte differentiation pathway and is required for expression of Sox5 and Sox6. Genes & Development 16, 2813–2828, doi: 10.1101/gad.1017802 (2002). 12414734 PMC187468

[b25] Hattori T. . SOX9 is a major negative regulator of cartilage vascularization, bone marrow formation and endochondral ossification. Development 137, 901–911, doi: 10.1242/dev.045203 (2010). 20179096

[b26] Tew S. R., Clegg P. D., Brew C. J., Redmond C. M. & Hardingham T. E. SOX9 transduction of a human chondrocytic cell line identifies novel genes regulated in primary human chondrocytes and in osteoarthritis. Arthritis Research & Therapy 9, 1–10, doi: 10.1186/ar2311 (2007). PMC2212576 17935617

[b27] Henry S. P., Liang S., Akdemir K. C. & Crombrugghe B. d. The postnatal role of Sox9 in cartilage. Journal of Bone and Mineral Research 27, 2511–2525, doi: 10.1002/jbmr.1696 (2012). 22777888 PMC3502666

[b28] Kim Y. . Generation of transgenic mice for conditional overexpression of Sox9. Journal of bone and mineral metabolism 29, 123–129, doi: 10.1007/s00774-010-0206-z (2011). 20676705 PMC3977853

[b29] Cucchiarini M. . Restoration of the extracellular matrix in human osteoarthritic articular cartilage by overexpression of the transcription factor SOX9. Arthritis Rheum 56, doi: 10.1002/art.22299 (2007). 17195218

[b30] Akiyama H. . Interactions between Sox9 and β-catenin control chondrocyte differentiation. Genes & Development 18, 1072–1087, doi: 10.1101/gad.1171104 (2004). 15132997 PMC406296

[b31] Ramaswamy G., Sohn P., Eberhardt A. & Serra R. Altered responsiveness to TGF-β results in reduced Papss2 expression and alterations in the biomechanical properties of mouse articular cartilage. Arthritis Research & Therapy 14, 1–15, doi: 10.1186/ar3762 (2012). 22394585 PMC3446415

[b32] Chavez. R.D., Coricor G., Perez J., Seo H.S & Serra R. Sox9 protein is stabiized by TGF-beta and regulates PAPSS2 mRNA in chondrocytes. Osteoarthritis and Cartilage doi: 10.1016/j.joca.2016.10.007 (2016). 10.1016/j.joca.2016.10.007 PMC5258840 27746378

[b33] Furumatsu T., Tsuda M., Taniguchi N., Tajima Y. & Asahara H. Smad3 Induces Chondrogenesis through the Activation of SOX9 via CREB-binding Protein/p300 Recruitment. Journal of Biological Chemistry 280, 8343–8350, doi: 10.1074/jbc.M413913200 (2005). 15623506

[b34] Kim G.-Y. . The stress-activated protein kinases p38 alpha and JNK1 stabilize p21(Cip1) by phosphorylation. The Journal of biological chemistry 277, 29792–29802, doi: 10.1074/jbc.M201299200 (2002). 12058028

[b35] Huang W., Zhou X., Lefebvre V. & de Crombrugghe B. Phosphorylation of SOX9 by Cyclic AMP-Dependent Protein Kinase A Enhances SOX9’s Ability To Transactivate aCol2a1 Chondrocyte-Specific Enhancer. Molecular and Cellular Biology 20, 4149–4158, doi: 10.1128/mcb.20.11.4149-4158.2000 (2000). 10805756 PMC85784

[b36] Zhao L., Li G. & Zhou G.-Q. SOX9 Directly Binds CREB as a Novel Synergism With the PKA Pathway in BMP-2–Induced Osteochondrogenic Differentiation. Journal of Bone and Mineral Research 24, 826–836, doi: 10.1359/jbmr.081236 (2009). 19113914

[b37] McMahon S., Charbonneau M., Grandmont S., Richard D. E. & Dubois C. M. Transforming growth factor beta1 induces hypoxia-inducible factor-1 stabilization through selective inhibition of PHD2 expression. The Journal of biological chemistry 281, 24171–24181, doi: 10.1074/jbc.M604507200 (2006). 16815840

[b38] Yang X. . TGF-β/Smad3 signals repress chondrocyte hypertrophic differentiation and are required for maintaining articular cartilage. J Cell Biol 153, doi: 10.1083/jcb.153.1.35 (2001). PMC2185521 11285272

[b39] Yang Y. . TRPV1 Potentiates TGFβ-Induction of Corneal Myofibroblast Development through an Oxidative Stress-Mediated p38-SMAD2 Signaling Loop. PLoS ONE 8, e77300, doi: 10.1371/journal.pone.0077300 (2013). 24098582 PMC3788725

[b40] Blaney Davidson E. N., Vitters E. L., van den Berg W. B. & van der Kraan P. M. TGF beta-induced cartilage repair is maintained but fibrosis is blocked in the presence of Smad7. Arthritis Res Ther 8, doi: 10.1186/ar1931 (2006). PMC1526625 16584530

[b41] Cucchiarini M., Terwilliger E. F., Kohn D. & Madry H. Remodelling of human osteoarthritic cartilage by FGF-2, alone or combined with Sox9 via rAAV gene transfer. J Cell Mol Med 13, doi: 10.1111/j.1582-4934.2008.00474.x (2009). PMC6512355 18705695

[b42] Park O.-J., Kim H.-J., Woo K.-M., Baek J.-H. & Ryoo H.-M. FGF2-activated ERK Mitogen-activated Protein Kinase Enhances Runx2 Acetylation and Stabilization. Journal of Biological Chemistry 285, 3568–3574, doi: 10.1074/jbc.M109.055053 (2010). 20007706 PMC2823497

[b43] Fuchs S. Y., Tappin I. & Ronai Z. e. Stability of the ATF2 Transcription Factor Is Regulated by Phosphorylation and Dephosphorylation. Journal of Biological Chemistry 275, 12560–12564, doi: 10.1074/jbc.275.17.12560 (2000). 10777545

[b44] Zhang Y. E. Non-Smad pathways in TGF-β signaling. Cell research 19, 128–139, doi: 10.1038/cr.2008.328 (2009). 19114990 PMC2635127

[b45] Seto H. . Distinct roles of Smad pathways and p38 pathways in cartilage-specific gene expression in synovial fibroblasts. The Journal of Clinical Investigation 113, 718–726, doi: 10.1172/JCI19899. PMC351321 14991070

[b46] Tew S. R. & Hardingham T. E. Regulation of SOX9 mRNA in Human Articular Chondrocytes Involving p38 MAPK Activation and mRNA Stabilization. Journal of Biological Chemistry 281, 39471–39479, doi: 10.1074/jbc.M604322200 (2006). 17050539

[b47] Ford-Hutchinson A. F. . Degenerative knee joint disease in mice lacking 3′-phosphoadenosine 5′-phosphosulfate synthetase 2 (Papss2) activity: a putative model of human PAPSS2 deficiency-associated arthrosis. Osteoarthritis Cartilage 13, doi: 10.1016/j.joca.2004.12.011 (2005). 15882565

[b48] Hellingman C. A. . In *Tissue Eng Part A* (2010).

[b49] Li T.-F. . Aberrant hypertrophy in Smad3-deficient murine chondrocytes is rescued by restoring transforming growth factor β–activated kinase 1/activating transcription factor 2 signaling: A potential clinical implication for osteoarthritis. Arthritis & Rheumatism 62, 2359–2369, doi: 10.1002/art.27537 (2010). 20506210 PMC2921996

[b50] Hayes S. A., Huang X., Kambhampati S., Platanias L. C. & Bergan R. C. p38 MAP kinase modulates Smad-dependent changes in human prostate cell adhesion. Oncogene 22, 4841–4850 (0000). 12894225 10.1038/sj.onc.1206730

[b51] Zhang M., Fraser D. & Phillips A. ERK, p38, and Smad Signaling Pathways Differentially Regulate Transforming Growth Factor-β1 Autoinduction in Proximal Tubular Epithelial Cells. The American Journal of Pathology 169, 1282–1293, doi: 10.2353/ajpath.2006.050921 (2006). 17003485 PMC1698849

[b52] Yu L., Hébert M. C. & Zhang Y. E. TGF-β receptor-activated p38 MAP kinase mediates Smad-independent TGF-β responses. The EMBO Journal 21, 3749–3759, doi: 10.1093/emboj/cdf366 (2002). 12110587 PMC126112

[b53] Han F. . Transforming growth factor-β1 (TGF-β1) regulates ATDC5 chondrogenic differentiation and fibronectin isoform expression. Journal of Cellular Biochemistry 95, 750–762, doi: 10.1002/jcb.20427 (2005). 15832361

[b54] Chen P., Vukicevic S., Sampath T. K. & Luyten F. P. Bovine Articular Chondrocytes Do Not Undergo Hypertrophy when Cultured in the Presence of Serum and Osteogenic Protein-1. Biochemical and Biophysical Research Communications 197, 1253–1259, doi: 10.1006/bbrc.1993.2612 (1993). 8280141

[b55] Pfaffl M. W., Horgan G. W. & Dempfle L. Relative expression software tool (REST©) for group-wise comparison and statistical analysis of relative expression results in real-time PCR. Nucleic Acids Research 30, e36, doi: 10.1093/nar/30.9.e36 (2002). 11972351 PMC113859

[b56] Zhai Z., Yao Y. & Wang Y. Importance of Suitable Reference Gene Selection for Quantitative RT-PCR during ATDC5 Cells Chondrocyte Differentiation. PLoS ONE 8, e64786, doi: 10.1371/journal.pone.0064786 (2013). 23705012 PMC3660368

