# Supplementary material for: TGF-β regulates phosphorylation and stabilization of Sox9 protein in chondrocytes through p38 and Smad dependent mechanisms
Source: Sci Rep. 2016 Dec 8;6:38616. doi: 10.1038/srep38616 (PMC5144132; doi:10.1038/srep38616)
Supplement: Supplementary Information [file srep38616-s1.pdf]

## Supplementary information for:

# TGF-β regulates phosphorylation and stabilization of Sox9 protein in chondrocytes through p38 and Smad dependent mechanisms.

**George Coricor and Rosa Serra***

University of Alabama at Birmingham, Department of Cell, Developmental, and Integrative Biology, Birmingham, Alabama, 35294-0005, USA.

* rserra@uab.edu

**Supplementary Table S1- Conserved Sox9 Serine 211 p38 phosphorylation motif**

| Species | Sequence |
|---|---|
| | 211 |
| Human | FKALQAD**S**PHSSSGM |
| Chimpanzee | FKALQAD**S**PHSSSGM |
| Mouse | FKALQAD**S**PHSSSGM |
| Bovine | FKALQAD**S**PHSSSGM |
| Platypus | FKALQAD**S**PHSSSGM |
| Chicken | FKALQAD**S**PHSSSGM |
| Frog | FKALQAD**S**PHSSSGI |

**Supplementary Table S2. Primer Sequences for qPCR**

| Gene | Forward Primer | Reverse Primer |
|---|---|---|
| *Mouse Sox9* | AGG AAG TCG GTG AAG AAC GG | GGA CCC TGA GAT TGC CCA GA |
| *Mouse Papss2* | CCC GTG ATG GAG TCA ACA TGA G | GTG CTT TGC AGT GGG TGT TCC |
| *Mouse PPIA*[56] | CGC GTC TCC TTC GAG CTG TTT G | TGT AAA GTC ACC CTG GCA CAT |

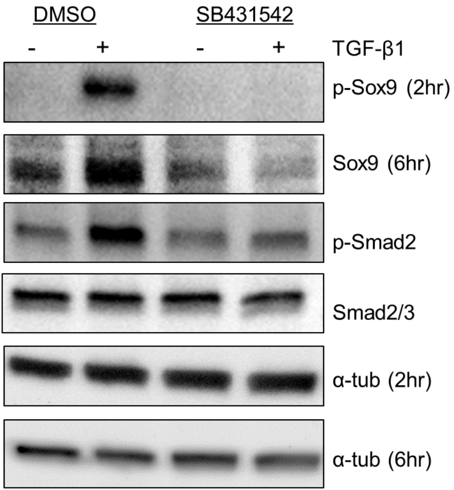


**Supplementary Figure S1: SB431542 blocks TGF-β1 mediated upregulation of p-Sox9 and Sox9 proteins.** ATDC5 cells were pre-treated with SB431542 or DMSO control for 2 hours, then treated with TGF-β1 or vehicle control for 2 or 6 hours. Cell lysates were collected and used for immunoblotting for p-Sox9, Sox9, p-Smad2, and Smad2/3. To ensure equal loading, α-tubulin was used as loading control.

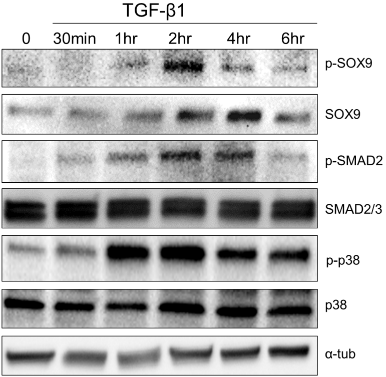


**Supplementary Figure S2: SOX9 phosphorylation and protein levels are up-regulated by TGF-β1 in bovine articular chondrocytes.** A) Bovine articular chondrocytes were treated with TGF-β1 for varying times as indicated at which point protein lysates were collected. SOX9, p-SOX9, SMAD2/3, p-SMAD2, p38, and p-p38 protein levels were determined by immunoblot. α-tubulin was used as a loading control. N=3.

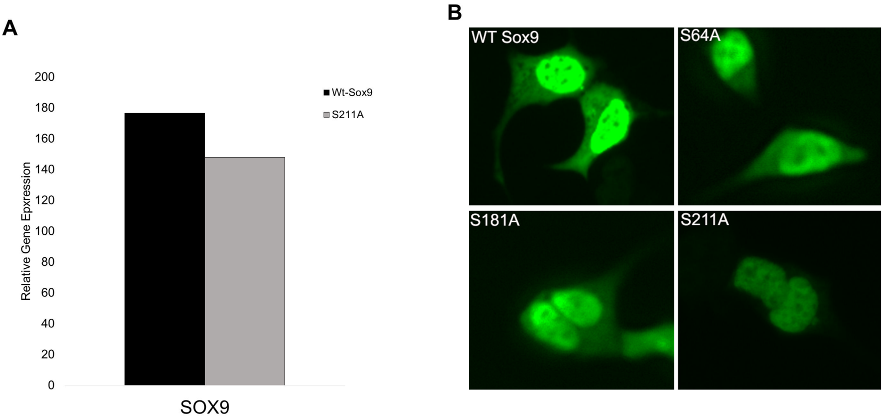


**Supplementary Figure S3: Sox9 S211A mutant mRNA is transcribed and protein can localize to the nucleus.** A) QPCR was used to show that the Sox9 S211A mutant was transcribed at similar levels to the WT-Sox9. B) HEK 293 cells were transfected with HA-tagged WT Sox9 or HA-tagged mutants (S64A, S181A, and S211A). Immunofluorescence staining was performed using anti-HA antibody and Alexa-Flour 488 conjugated secondary antibody (green). Representative fluorescence images show Wt-Sox9 and Sox9 mutants are able to translocate to the nucleus. No signal was observed in samples incubated with secondary antibody alone (not shown).

**Supplementary Figure 4-1 to 10: Examples of uncropped western blots.**

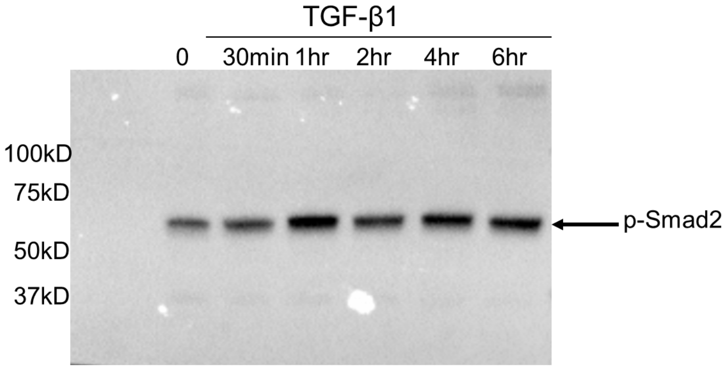


Example of p-Smad2 uncropped western blot, from Figure 1D

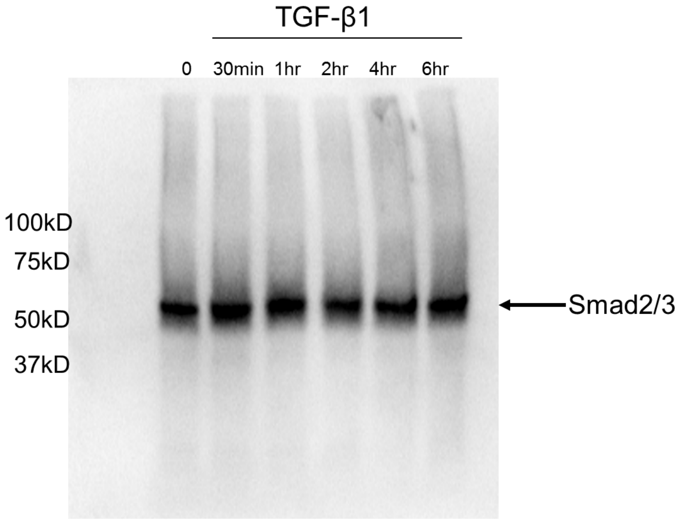


Example of Smad2/3 uncropped western blot, from Figure 1D

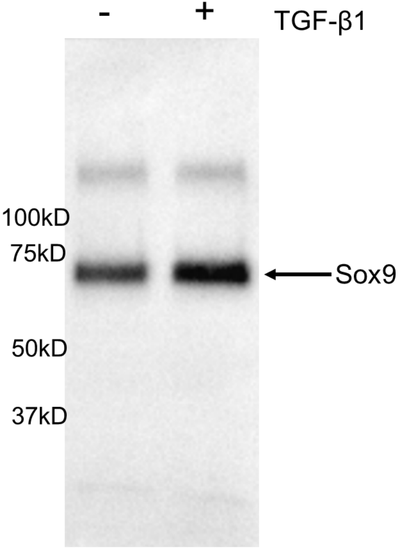


Example of Sox9 uncropped western blot, from Figure 1B

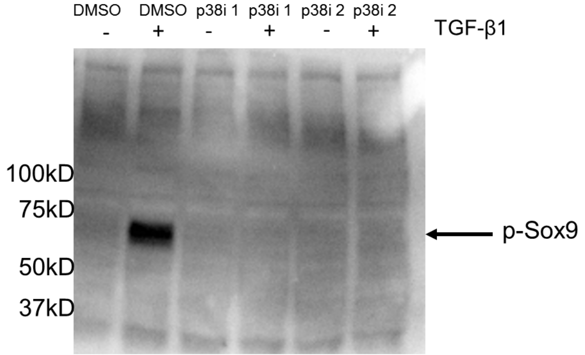


Example of p-Sox9 uncropped western blot, from Figure 3C

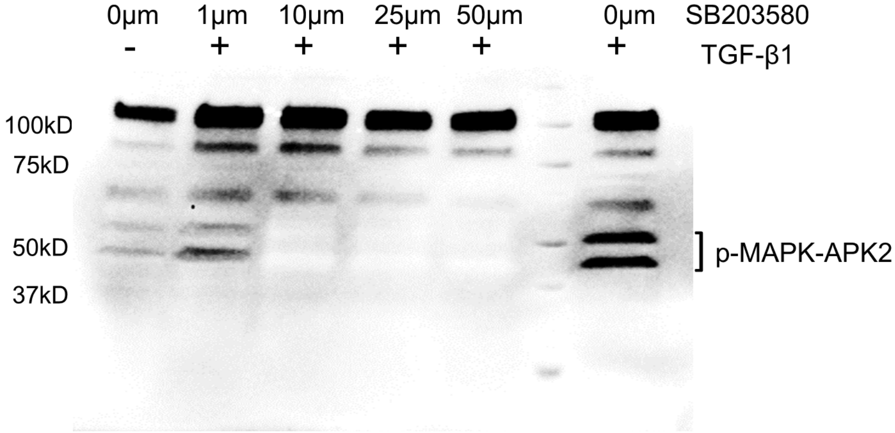


Example of p-MAPK-APK2 uncropped western blot, from Figure 3A

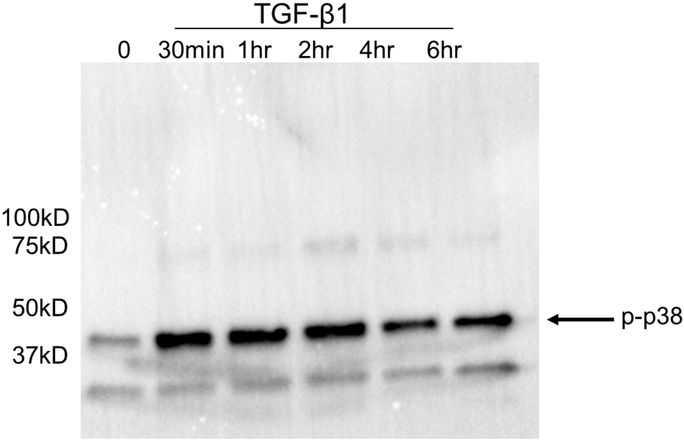


Example of p-p38 uncropped western blot, from Figure 1D

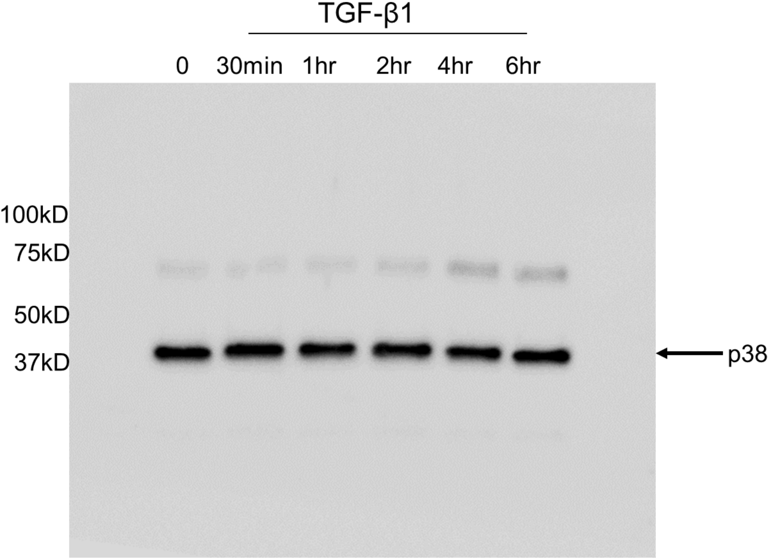


Example of p38 uncropped western blot, from Figure 1D

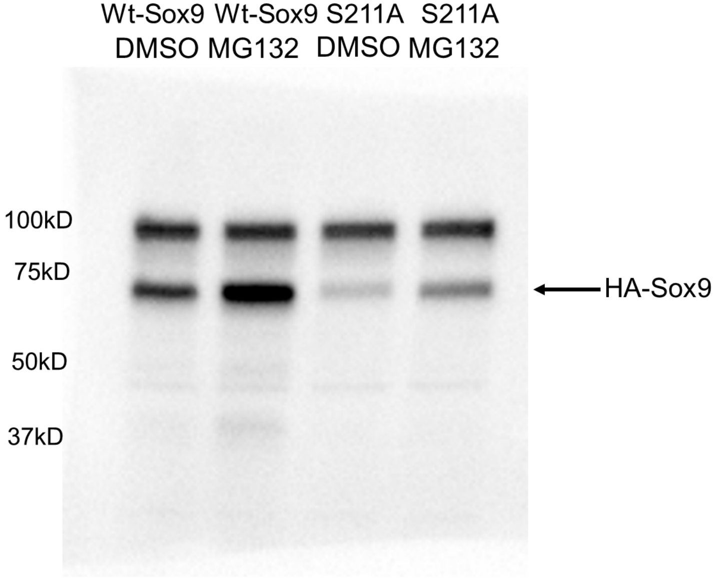


Example of HA-Sox9 uncropped western blot, from Figure 2C

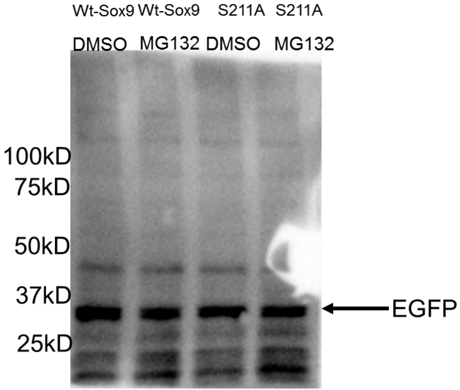


Example of EGFP uncropped western blot, from Figure 2C

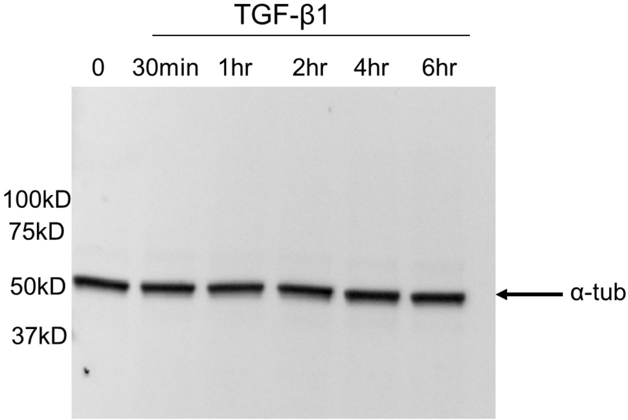


Example of α-tub uncropped western blot, from Figure 1D
